# Maternal distress, parenting stress, maladaptive parenting and children’s problematic media use in China: evidence from 2022 spring in Shanghai

**DOI:** 10.1186/s12889-024-19382-0

**Published:** 2024-07-11

**Authors:** Jingyao Wang, Shumin Wang, Bowen Xiao, Juan Li, Yuemin Feng, Yan Li

**Affiliations:** 1 Shanghai Institute of Early Childhood Education, Shanghai Normal University, 100 Guilin Road, Xuhui District, Shanghai, China; 2 Carleton University, Ottawa, Canada

**Keywords:** Maternal COVID-19 distress, Parenting stress, Maladaptive parenting, Children’s problematic media use

## Abstract

The COVID-19 lockdown has forced young children to spend more time on media and significantly impacted their mothers’ mental health. This study explored how mothers’ individual distress influences children’s problematic media use during the Shanghai citywide lockdown caused by COVID-19. Data were collected from 1889 Chinese mothers (*M*_age_ = 34.69 years, *SD* = 3.94 years) with preschoolers aged 3–6 years (*M*_*ag*e_ = 4.38 years, *SD* = 1.06 years; 49.0% boys) via an online survey. The statistical analyses relied on SPSS Statistics version 26.0 and macro-program PROCESS 3.3. to investigate the associations and mediation analysis among all the study variables. The results indicated a positive association between maternal distress and children’s problematic media use, mediated by parenting stress and maladaptive parenting. Specifically, the serial mediation analysis revealed that high levels of maternal distress exacerbate parenting stress, which in turn leads to maladaptive parenting practices. These maladaptive practices subsequently increase problematic media use in preschool children. The findings highlighted that parents need to enhance their ability to manage risk and promote mental health during periods of significant stress and routine disruption to reduce children’s problematic media use.

Excessive media use has increased during the COVID-19 lockdowns in response to remote working, online learning, social interaction, and entertainment [1, 2], which makes the significant increase in global children’s screen time an important public health problem [3, 4]. Several studies have recently focused more on the influencing factors of excessive media use [5, 6] during COVID-19 lockdowns, and problematic media use (PMU) is less focused on in Chinese preschool children. Nevertheless, it is important to distinguish between excessive media use and PMU [7], as there is no conclusive evidence to suggest that screen time alone has a direct negative impact on the psychosocial development of children [8]. Screen time is also known as screen exposure time, which refers to the time spent by an individual on a series of activities related to screen electronic media [9]. Excessive media use refers to consuming media in quantities beyond what is considered healthy or balanced, but it is not necessarily always harmful. Excessive media use refers to consuming media in unhealthy quantities, and screen time is a broader term encompassing all activities involving screens, which can become problematic if excessive. Moreover, problematic media use focuses on the harmful effects of media consumption on individual developmental functioning. According to Domoff et al. [7], individual impaired functioning is critical in distinguishing screen exposure/excessive use from a problematic level of media use. Thus, we focus on the problematic media use of preschool children and go beyond the literature that mainly focuses on the influence of general parenting styles [10] and consider COVID-19-related parental distress and parenting stress.

## Problematic media use in childhood

Increasing numbers of children are growing up in households with media technologies, and they spend an amount of time on different devices [11], exceeding the recommendation of the World Health Organization (WHO) that children under 2 years old should avoid using screens, and children aged 2–5 should not exceed 1 hour per day [12]. According to the 51st Statistical Report on China’s Internet Development published in March 2023, the number of Internet users in China had reached 1.067 billion by December 2022, with 4.4% being children under the age of ten [13]. Research has found that by age 4 years, between one-third and three-quarters of children have access to their own mobile devices [14, 15]. Radesky et al. [15]found that younger children are acquiring their first mobile devices, and using them for several hours a day. According to the displacement theory, excessive media use may replace time spent participating in activities critical for children’s social-emotional development, particularly activities involving physical movement and interpersonal communication, and may thus harm children’s mental health and well-being [16, 17].

Problematic media use (PMU) in childhood has been defined as excessive use leading to dysfunction in a major domain of a child’s development: social, behavioral, or academic [18]. Individuals must experience conflict or disturbance in psychosocial functioning to indicate a severe problem with screen media use, not just the number of hours used [18]. As Bronfenbrenner [19] proposed, children develop within a series of environmental systems that interact with each other. In other words, children do not grow or develop in a vacuum—parents and other caregivers, peers, and technology all exert influence on children’s growth and development. Based on Bronfenbrenner’s conceptualization, Domoff et al., [7] incorporate social learning and behavioral principles (e.g., reinforcement) to propose the Interactional Theory of Childhood Problematic Media Use (IT-CPU), which showed that household chaos or lack of routines, structure in the home [20], and parental stress have effects on children’s PMU and also pointed that parents’ perception of stress levels likely contribute to greater PMU via using screens as a behavior management tool [21]. Therefore, we expected that family dynamics would play a significant role in shaping children’s PMU.

### Parental distress during the COVID-19 lockdown

The COVID-19 pandemic was a remarkable occurrence that emerged unpredictably and raised a global public health issue. At the end of 2019, China received official reports of the first COVID-19 cases [22]. Cases in Shanghai began rising in March 2022, soon spiraling into the worst flare-up the country has seen since the initial outbreak in Wuhan in early 2020 [23]. From the end of February to May 30th, COVID-19 caused 6 hundred thousand people to be impacted, and Shanghai introduced a staggered lockdown in late March. This had expanded into a full citywide lockdown by early April that is keeping most of its 25 million residents, whether urban or rural, trapped at home [24], which is called a “super frozen city”. Alongside concerns about financial security, people had their daily routines disrupted indefinitely and were isolated from people and places that were part of their daily lives [25]. These changes seriously affected people’s mental health, especially psychological distress [26]. Therefore, investigating the COVID-19 lockdowns’ impact on parents and children represents a relevant and impactful issue.

Based on the study conducted by Kazak et al., [27] and Li et al., [28], parental distress associated with COVID-19 may be divided into two primary aspects: parents’ subjective overall distress connected to COVID-19 events and parents’ perceived distress of children. Both aspects are connected to the stress encountered by parents during the lockdown: the former general psychological distress caused by various stressors (e.g., financial challenges, concern for family members, and struggle to access food or medical care), as well as specific distress associated with taking increased parenting duties and responsibilities compared to before (e.g., paying attention to children’s physical and mental health, attending to children’s daily needs and managing online learning). According to Babore et al., [29], general psychological distress is caused by factors beyond childrearing, such as the broader social and environmental context, including routine obligations and responsibilities [30]. The specific distress is connected with parental role and parenting. In the context of COVID-19, the pandemic has affected the division of housework, parent-child relationship, and the physical and mental health of children and family members [31, 32] and brought significant psychological burden and distress to families [33], especially mothers [34]. During the epidemic, Chinese mothers reported more severe psychological distress and stress symptoms than before [35].

### Parental distress and child problematic media use

Previous studies have found that maternal poor mental health (such as distress or depression) is associated with increased screen time among young children [36–38]. Mental health problems might make mothers use media as an ‘electronic babysitter’ to compensate for their lack of energy and fatigue, leading to women’s sedentary and increased frequency of using their phones, thus unintentionally increasing their children’s media exposure [39]. Distressed mothers are less successful in monitoring their children’s behaviors [39] and allow more interruptions from devices in their parent-child interactions [40], increasing the risk of children’s PMU. Because of parenting demands, parents in more chaotic environments and with greater stress levels may be less likely to supervise and manage their children’s media use [20]. Meanwhile, children from more chaotic homes and higher parenting stress have greater externalizing problem behaviors [41], and parents may be more likely to use media as a pacifying device [42]. Several studies suggested maternal distress or depression was positively associated with children’s excessive screen time [37, 38]. Thus, it is possible that parents who reported social disruption due to COVID-19 experienced heightened levels of distress [43], and parents may have less control over their children’s ability to maintain healthy behaviors in this circumstance [44]. On these grounds, it seems reasonable to hypothesize that COVID-19 maternal distress would be positively associated with children’s PMU.

### Parental distress and child problematic media use: the mediating role of parenting stress

Parenting stress is distinct from stress in other realms of life (for example, work stress and marriage relationship stress). It refers to the bad experience of parents as a result of a perceived discrepancy between their parental responsibilities and available resources [45, 46]. Notably, the lockdown can cause isolation-related psychological distress for caregivers [32], especially for women [47, 48], which is associated with parenting stress [49]. According to the stress process model, the stressor pile-up caused by the crisis event (e.g., COVID-19) and additional stressors that occur over time (e.g., home isolation, work at home, child rearing and lack of family materials) created secondary stressors (e.g., parenting stress) [50]. Furthermore, when affected adults have young children, their ability to fulfill the parenting role is impaired, exacerbating the negative impact on children [51, 52]. The spillover hypothesis theory suggests the emotional or behavioral patterns of family members in one context may shift to another in a family system [53], meaning that the perceived stress (epidemic-related stress or stressors) from parents may spill over into parenting.

Besides, IT- CPU indicates that high parenting stress levels [54] also correspond to a longer duration of children’s PMU, firstly because parents use mobile devices to avoid uncomfortable family situations [55], this casually teaches children that using media as a self-regulation strategy is possible [7]. Children learn to imitate their parents’ behaviors at a young age [56]; it is likely that children learn about technology by observing their caregivers’ media use. Second, digital media can be a resource for child care in low-support settings [57]; parents may feel compelled to provide digital media when confronted with unreasonable and stressful demands or excessive parenting stress [58, 59]. As a result, we hypothesized that maternal parenting stress would independently mediate the association between COVID-19 maternal distress and children’s PMU.

### Parental distress and child problematic media use: the mediating role of maladaptive parenting practices

Parenting practices are the observable actions that parents take about their children to help them participate in social activities and achieve goals [60, 61]. In this study, we focused on maladaptive parenting practices (including high levels of hostility, punishment, criticism, and proclivity) in the context of parents’ responses to stressful events. Experiences with COVID-19 distress may lead to parental maladaptive parenting practices. According to the general strain theory [62] and a social-ecological perspective [63], Pereda and Díaz-Faes [64] proposed that the cumulative effect of COVID-19 stressors can exacerbate parental anger and distress-increasing risk of intra-familial turmoil and potential for maladaptive parenting practices. It is proposed that maladaptive parenting comes in response to experienced distress, with parents displaying dysfunctional emotion-focused coping behaviors towards the child, behaviors that appear as yelling, insulting, threatening, and punishing [65].

Maladaptive parenting seemed to be a risk factor for mental disorders, more dysfunctional gaming, and sleep disorders [66], which may cause high levels of parent-child conflict and parental psychological control associated with high levels of media use and addiction in adolescents [67, 68]. Furthermore, studies have shown that maladaptive parenting is associated with high media usage time [10] in school-aged children and internet addiction [69] in secondary school students. Moreover, children and adolescents from maladaptive parenting have higher scores of symptoms of gaming addiction [66]. Based on what has been discussed, we hypothesize that maladaptive parenting would mediate the relationship between maternal COVID-19 distress and children’s PMU in preschoolers.

### The serial mediating roles of parenting stress and maladaptive parenting practices

In particular, we assume that the two mediators – parenting stress and maladaptive parenting – might co-play a serial mediating role in the relationship between COVID-19 maternal distress and children’s PMU. The parental stress model [70] describes that parenting stress mediates the impact of environmental stresses on parenting outcomes such as harsh parenting and parent-child relationships. People’s mental health and general well-being have suffered as a result of the lockdown policy, stay-at-home orders, and other measures [26, 71], which may exacerbate parenting stress [72, 73]. When under a lot of parenting stress, parents will transfer their negative emotional experience to maladaptive parenting practices [74, 75], which leads to child maladjustment, such as externalizing problems [76] and internalizing symptoms [77].

In the context of COVID-19, parenting stress was usually related to parent-child interaction because both children and parents engage in various activities at home, such as providing all-day childcare while dealing with online education requirements, managing remote offices, and balancing the needs of many family members [51, 78]. According to research, when children are not in school, they are more bored, less physically active, and spend more time in front of screens [79], which may raise the possibility of conflict with parents and lead to parenting stress and maladaptive parenting. Since parents experienced multiple challenges directly related to parenting during the lockdown [78, 80], we speculated a serial mediation model in which parental distress influences children’s PMU indirectly, through parenting stress and maladaptive parenting.

### The context of this study

Shanghai has implemented a citywide lockdown since April 1, 2022, with all adults and children trapped at home for about 3 months and conducting multiple rounds of universal nucleic acid tests, which were very special and unique prevention and lockdown measures worldwide. During the lockdown, families’ living conditions changed dramatically and abruptly. In the home, the words, practices, and responsibilities of parents become more important than usual. Parents, on the other hand, had been left to manage homeschooling, lifestyle and behavioral habits, and childcare in unprecedented ways.

Accordingly, this study is dedicated to understanding the underlying mechanisms between maternal distress and PMU in Chinese preschoolers. Our theoretical background to this examination stems from the IT-CPU, reflecting household chaos or lack of routines and parenting stress that influence children’s PMU [7]. It is, therefore, important to consider the role of the broad family environment and parental factors to fully understand the determinants of PMU.

**Fig. 1:**
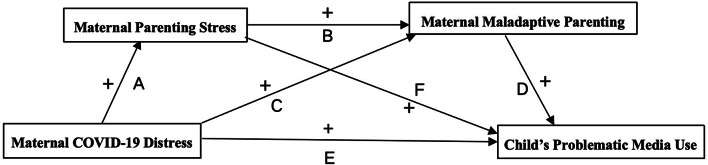
Proposed model for this study. *Note “+ −” = positive or negative effect; A-F = paths*

Based on previous research, we proposed that maternal distress was positively associated with children’s PMU (H1), that maternal parenting stress would independently mediate the relationship between maternal distress and children’s PMU (H2), and that maladaptive parenting could mediate the relationship between maternal distress and children’s PMU (H3). Moreover, we also speculate that maternal distress influences child PMU indirectly through parenting stress and maladaptive parenting (H4). Our conceptual model of the current study is shown in Fig. 1.

## Method

### Participants

Participants were *N* = 1889 mothers (*M*_age_ = 34.69 years, *SD* = 3.94 years) with children aged 3–6 years (*M*_*ag*e_ = 4.38 years, *SD* = 1.06 years; 49.0% boys) from several kindergartens in urban areas in Shanghai, People’s Republic of China. Almost all children were Han Chinese, a predominant ethnic group (over 91.51% of the population) in China. Participants are all media users and their characteristics are presented in Table 1.

**Table 1:** Demographic characteristics of participants

Variables	Groups	*N* (%)
Age of Child	Age 3	431 (22.8)
	Age 4	721 (38.2)
	Age 5	323(17.1)
	Age 6	414 (21.9)
Gender of Child	Girl	964 (51.0)
	Boy	925 (49.0)
Age of Mother	≤ 30 years	248 (13.1)
	31–35 years	983 (52.0)
	36–40 years	499 (26.4)
	41–45 years	143 (7.6)
	≥ 46 years	16 (0.8)
Mother Education Level	High school or below	92 (4.9)
	College/Associate degree	290 (15.4)
	Bachelor degree	1156 (61.2)
	Postgraduate degree or high	351 (18.6)

*Note* N = Number; % = the percentage of the total number of participants

### Procedure

The study informs participants of the study’s goals, and that participation is voluntary and can be withdrawn at any time. To prevent the spread of Severe Acute Respiratory Syndrome Coronavirus 2 through droplets or contact, the recruitment letter and the questionnaires were released on Wenjuanxing (www.wjx.cn), the largest online survey platform in China. This online survey was distributed via the WeChat public platform and data were collected from May 1 to June 10, 2022. All Chinese users of WeChat and other social media platforms are welcome to view this survey and respond by scanning the QR code on the survey’s address or clicking the appropriate link. There are no personal identifiers stored or recorded in this study. Thus, there are no potential privacy risks. After completing the data collection, we used the information provided by the mothers to create evaluation results in the form of charts and text. These results, along with targeted family education suggestions, were sent to their email addresses. Our goal was to help mothers better understand their family environment and their child’s development.

### Measure

#### Maternal COVID-19 distress

The Distress Scale from the COVID-19 Exposure and Family Impact Survey (CEFIS) [27] uses two items to assess how much distress caregivers have experienced due to the pandemic [81]. In the prior study, internal consistency was good for (α = 0.76) the Distress scale [27]. The first item asks the parent to rate how much distress they have experienced, and the second reflects the parents’ perception of how much distress the children in their family have experienced during the lockdowns. Both items use a 10-point scale. The two items were translated from English into Mandarin and back-translated into English, with discrepancies between versions resolved by bilingual faculties and graduated students from the psychology department at *Shanghai Normal University*. According to Eisinga et al., [82], the Spearman-Brown coefficient and the standardized alpha coefficient were the most reliable statistics for the two-item scale. In our study, Cronbach’s α for the CEFIS Distress Scale was 0.82, and the Spearman-Brown coefficient was 0.82.

#### Maternal parenting stress

Mothers completed the Chinese version of the Parenting Stress Index-Short Form (PSI-SF) developed by Abidin et al., [83]. This measure has demonstrated evidence of reliability and validity among parents of Chinese children [84]. The PSI-SF has 36 items pertaining to parental feelings and experiences in three dimensions: Parental Distress (PD; 12 items; e.g., “I often feel I cannot deal with things very well”), Parent-child Dysfunctional Interaction (PCDI; 12 items; e.g., “My child rarely does anything that makes me feel good”), and Difficult Child (DC; 12 items; e.g., “My child seems to be more of a cry baby than the most child”). Each subscale contains 12 items. The total PSI-SF score is considered an indicator of the parents’ overall experience of parenting stress. In the current study, Cronbach’s alpha values were 0.96 for the PSI-SF total score.

#### Maternal maladaptive parenting

Mothers completed the Chinese version of the Parenting Styles and Dimensions Questionnaire [85, 86]. This version of the PSDQ contained 26 items forming two stylistic patterns of parenting: adaptive parenting and maladaptive parenting [87]. Of particular interest for the present study were the subscales assessing maternal maladaptive parenting which consisted of 3 subscales: physical punishment (5 items; e.g., “I use physical punishment as a way of disciplining our child”), verbal hostility (3 items; e.g., “Yells or shouts when child misbehaves”), non-reasoning (3 items; e.g., “Punishes by taking privileges away from child with little if any explanations”). In the current study, this measure demonstrated high internal reliability (e.g., Cronbach’s alpha values were 0.90).

#### Problematic media use

Mothers completed the Chinese version of the Problematic Media Use Measure scale (PMUM) developed by Domoff et al. [18]. This measure has demonstrated evidence of reliability and validity among parents of Chinese children [88]. The Chinese version of PMUM consists of 23 items and two subscales: psychosocial problems (11 items; e.g., “My child’s screen media use interferes with family activities”) and tolerance & withdrawal (12 items; e.g., “It is hard for my child to stop using screen media”). Each item was rated on a 5-point Likert scale (1 = “never” to 5 = “always”). Higher scores indicate a higher level of PMU. In this study, Cronbach’s α for tolerance and preoccupation and psychosocial functioning were 0.94 and 0.96.

### Statistical analyses

First, we adopted the Harman single-factor method to examine the common method bias due to the questionnaire’s self-report. According to the test results, there were 16 factors with characteristic roots of more than 1 in principal component analysis, and the variation explained by the first factor was 22.94%, which was lower than the critical value of 40%, demonstrating that there was no serious common method bias in this study. Second, Pearson correlation analyses were conducted to investigate the associations among all the study variables and to investigate multicollinearity. The high correlation between independent variables leads to the occurrence of multicollinearity. Third, to ensure the reliability of the model, a multicollinearity test was conducted. The multicollinearity test is used to determine whether and to what extent the independent variables under observation are correlated. The occurrence of a high degree of correlation is certainly a significant methodological problem, because independent variables thus lose their presumption of independence as one of the most important preconditions for their use. Fourth, mediation analysis was constructed to examine the hypothesized mediation models based on variables’ correlations, a regression-based approach. The collected data were analyzed using SPSS Statistics version 26.0 and macro-program PROCESS 3.3.

## Results

### Preliminary analyses

Means, standard deviations, and correlations among variables are presented in Table 2. Among the results, maternal COVID-19 distress was significantly and positively associated with maternal parenting stress (*p* < .001) and maladaptive parenting (*p* < .001). Then, parenting stress was significantly and positively associated with maladaptive parenting (*p* < .001). At last, children’s PMU was significantly positively correlated with maternal COVID-19 distress (*p* < .001), maternal parenting stress (*p* < .001), and maladaptive parenting (*p* < .001).

**Table 2:** Descriptive statistics and correlations for study variables

	1	2	3	4	5	6	7	8
1. Mother Age	1							
2. Mother’s Education	0.07**	1						
3. Child Gender	0.02	− 0.01	1					
4. Child Age	0.22***	0.06*	− 0.01	1				
5. Maternal Distress	0.02	− 0.07**	0.01	0.07**	1			
6.Parenting Stress	− 0.04	− 0.07**	0.04	− 0.11***	0.34***	1		
7.Maladaptive Parenting	− 0.03	− 0.04	0.10***	− 0.05*	0.26***	0.60***	1	
8.Problematic Media Use	0.01	− 0.04	0.07**	− 0.02	0.28***	0.54***	0.57***	1
*M*	-				3.71	1.92	1.92	1.87
*SD*	-				2.35	0.61	0.61	0.70

*Note* M = Mean; SD = Standard Deviation

* *p* < .05; ** *p* < .01; *** *p* < .001

### Mediation analyses

**Fig. 2:**
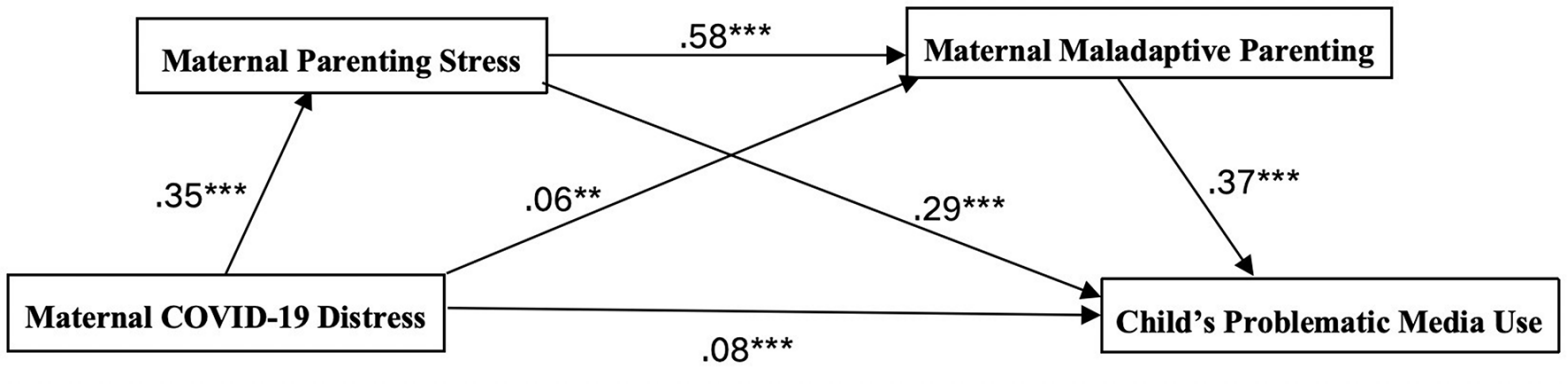
Results of serial mediational model. *Note* **p < .01; ***p < .001

The multicollinearity test indicated that the VIF (variance inflation factor) values of all independent variables were less than 3, indicating that there is no multicollinearity problem in the model. This meant that there was no highly correlated linear relationship between the variables, thus ensuring the stability of the model. Mediation analyses aimed to examine relations among maternal COVID-19 distress, maternal parenting stress, maladaptive parenting, and children’s PMU. Table 3; Fig. 2 show the results of the hypothesis testing. Confirming Hypothesis (1), the study found maternal COVID-19 distress was positively related to children’s PMU without any mediators. As shown in Table 4, the total effect of maternal COVID-19 distress on children’s PMU (total effect = 0.28, 95% CI = [0.24, 0.32]) was positive and significant.

**Table 3:** Direct and indirect effect maternal distress on children’ s problematic media use

Predictors	Model 1 (Outcome: PS)			Model 2 (Outcome: MP)			Model 3 (Outcome: PMU)		
	β	SE	t	β	SE	t	β	SE	t
Mother Age	0.00	0.00	− 0.91	0.01	0.00	-1.95	0.00	0.00	0.04
Mother’s Education	− 0.05	0.03	-1.75	0.01	0.03	0.45	0.00	0.03	0.08
Child Gender	0.08	0.04	1.89	0.13***	0.04	3.55	0.05	0.04	1.40
Child Age	− 0.12***	0.02	-5.95	0.01	0.02	0.74	0.02	0.02	1.26
Distress	0.35***	0.02	16.08	0.06***	0.02	3.23	0.08***	0.02	4.19
PS				0.57***	0.02	29.09	0.29***	0.02	12.43
MP							0.37***	0.02	16.42
PMU									
*R* ^*2*^	0.14			0.37			0.39		
*F*	59.64***			181.87***			172.24***		

*Note* Child gender was coded as 1 = girl and 2 = boy; Distress = Maternal COVID-19 Distress; PS = Parenting Stress; MP = Maladaptive Parenting; PMU = Problematic Media Use

*** *p* < .001

**Table 4:** Mediating effects of parenting stress and maladaptive parenting on the relationship between maternal distress and children’s problematic media use

Path	β	SE	95% Confidence interval	
			BootLLCI	BootULCI
Total indirect effect	0.20	0.02	0.17	0.23
Indirect effect: Distress→PS→PMU	0.10	0.01	0.08	0.12
Indirect effect: Distress→MP→PMU	0.02	0.01	0.01	0.04
Indirect effect: Distress→PS→MP→PMU	0.07	0.01	0.06	0.09
Total effect	0.28	0.02	0.24	0.32
Direct effect	0.08	0.02	0.04	0.12

*Note* Distress = Maternal COVID-19 Distress; PS = Parenting Stress; MP = Maladaptive Parenting; PMU = Problematic Media Use

Confirming Hypothesis (2), Table 3 (Model 1 and Model 3) showed that maternal COVID-19 distress was positively related to parenting stress, and parenting stress was positively related to children’s PMU. The study found maternal parenting stress mediated the relationship between maternal COVID-19 distress and children’s PMU (maternal COVID-19 distress → parenting stress → children’s PMU: effect = 0.10, SE = 0.01, 95% CI = [0.08, 0.13]) (see Table 4). In addition, Table 3 (Model 2 and Model 3) indicated maternal COVID-19 distress was positively correlated with maladaptive parenting, and maladaptive parenting was positively correlated with children’s PMU. It indicated that maladaptive parenting also mediated the relationship between maternal COVID-19 distress and children’s PMU (maternal COVID-19 distress → maladaptive parenting → children’s PMU: effect = 0.02, SE = 0.01, 95% CI = [0.01, 0.04]), also confirming Hypothesis (3), as seen from the results in Table 4. Finally, confirming Hypothesis (4), the study found maternal parenting stress and maladaptive parenting serial mediated the relationship between maternal COVID-19 distress and children’s PMU. Table 3 (Model 2) showed that parenting stress was positively associated with maladaptive parenting. The relationship was significant (maternal COVID-19 distress → parenting stress → maladaptive parenting → children’s PMU: effect = 0.07, SE = 0.01, 95% CI = [0.06, 0.09]), as shown in Table 4. To summarize, the study revealed a positive relationship between maternal COVID-19 distress and children’s PMU. This association was partially mediated by maternal parenting stress and parenting practices.

## Discussion

In the current study, we tested a conceptual model that explored mediating (i.e., parenting stress and maladaptive parenting) factors in the link between maternal distress and children’s PMU in Mainland China during COVID-19. Our results indicated maternal distress was positively associated with children’s PMU during COVID-19 lockdowns. Moreover, maternal distress was not only directly associated with children’s PMU but also indirectly related to children’s PMU via maternal parenting stress and maladaptive parenting.

### Maternal distress and children’s problematic media use

The degree to which parents can effectively limit their young child’s screen time may depend on parents’ mental health [89]. The current study found maternal COVID-19 distress was positively associated with children’s PMU. The findings are consistent with previous Chinese studies conducted before COVID-19 [38, 90], namely that the worse maternal mental health, the more children’s screen time reported. Mothers, who are often the primary caregivers [91], may experience increased distress during the lockdowns [92], making it difficult to manage children’s screen time. Tang et al., [93] found that mothers’ general stress was inversely associated with both monitoring and limiting children’s screen time. In addition, children during the pandemic-related quarantine were more likely to have problem behaviors [94, 95], increasing the child’s demands on the parent, which in turn exacerbates parental distress. When parents find it difficult to cope, they tend to use screen media as a “babysitter” to reduce child problem behavior and parent-child conflict [96]. According to Nabi and Krcmar [97], letting children use media can help parents reduce their own stress. This may unintentionally lead children to use media as a self-regulation strategy [7], which is not conducive to the development of children’s self-regulation abilities. Individuals with lower self-regulation capabilities may exhibit heightened sensitivity to external stimuli, such as media content, and encounter challenges in exerting control over and managing their media use [98, 99].

### The mediating role of parenting stress

Our findings showed that parenting stress independently mediated the relationship between maternal COVID-19 distress and children’s PMU. This finding lends support to the stress process model, which predicts that stressor accumulation causes maternal psychological distress and parenting stress [50, 100]. Furthermore, the findings are consistent with previous research showing that when parents have poor mental health, they report more parenting stress [51, 101], which also supports the spillover hypothesis theory. For example, Babore et al. [29] found that parenting stress plays an important mediating role between maternal distress and children’s problem behaviors. Parents in a poor psychological state experience increased parenting stress, indicating that general psychological distress can generate or worsen parenting stress. However, mothers who experience higher levels of parenting stress are more likely to require resources to help them reduce the demands of parenting [102]. During the lockdown, electronic media was the most convenient and available resource. Occupying their children with using media can provide parents with time to cope with parenting stress, potentially lowering the demands of parenting [102]. Furthermore, according to IT-CPU, mothers with higher parenting stress are more likely to use media to withdraw from tense family interactions and get a short rest [7]. In conclusion, the results show that the mothers perceived COVID-19 distress related to stress events exacerbated parenting stress, increasing the risk of children’s PMU. It is critical for parents to manage and cope with the major stressors, as well as regulate their mental health and relieve parenting stress, which reduces children’s PMU.

### The mediating role of maladaptive parenting

The findings of this study indicated that maladaptive parenting independently mediated the relationship between maternal COVID-19 distress and children’s PMU. As previously stated, the COVID-19 pandemic presented unexpected challenges for parents [103]; given that mothers in China may be more responsible for family care than fathers, these challenges have significantly increased maternal distress [104]. Parents with high levels of psychological distress are inclined to use punitive and harsher parenting practices with their children [105, 106], consistent with the general strain theory and positively predicts children’s PMU. This finding lends support to previous studies on adolescents [10, 69], indicating that maladaptive parenting practices are associated with excessive media use. When parents engage in maladaptive parenting practices and do not care about or spend time with their children, their children are more likely to seek emotional and psychological comfort through excessive use of media. Furthermore, maladaptive parenting will obstruct children’s psychological development [107, 108], eventually leading to PMU.

### The serial mediating roles of parenting stress and parenting practices

In this study, maternal parenting stress and parenting practices were found to sequentially mediate this relationship. Specifically, maternal COVID-19 distress was linked to maladaptive parenting via maternal parenting stress, exacerbating the child’s PMU. Our findings supported the parental stress model [70], demonstrating the impacts of environmental stresses on maladaptive parenting through parenting stress. Notably, higher levels of cumulative risk were associated with higher levels of psychological distress [32]. Maternal higher COVID-19 distress and mental health problems as a major family member will lead to higher parenting stress [109] and will transfer their negative emotional experience to maladaptive parenting [75], which may show less acceptance and encouragement of children. Instead, it is easier to engage in maladaptive parenting practices such as harsh and punitive measures [105, 106], exacerbating the children’s PMU.

### Limitations and future directions

The current study’s findings add to our understanding of the negative impact of stressful events (the COVID-19 pandemic) on Chinese parents and will serve as a reference for family-based practices in the event of possible pandemics in the future. It appears to be the first study to demonstrate that the links between COVID-19 distress and PMU in Chinese preschoolers are caused by parenting stress and parenting practices. Despite the fact that the results add to the existing literature, there are several limitations to consider when interpreting the findings.

First, the current study’s correlational design limits our ability to investigate causal links and the direction of effects between variables. It is suggested that future research use a longitudinal design to investigate those mediation effects and track the directional or transactional processes over time. Furthermore, all of the instruments used in this study were self-reported, which could lead to judgmental biases and shared method variances. Future research should collect data from multiple sources to obtain convergent evidence with alternative-source ratings of parenting practices (e.g., observations). Second, the data was obtained solely during COVID-19, and no data on any of the measures existed before the pandemic. Thus, it is difficult to determine whether the findings are unique to the COVID-19 context and whether there was an upsurge in PMU and use following the pandemic’s onset. Hence, cautious thought must be applied when applying the results to media use during routine times. In addition, the study does not consider the variation in the impact of COVID-19 by residence area, indicating a sampling bias. The impact of economic status on parents’ mental health and parenting styles [110] has not yet been considered. When exploring the impact of future public health emergencies in the future, it is necessary to consider regional distribution and changes in economic status. Third, two items were used to assess maternal COVID-19 distress, which is relatively simple but not sufficiently accurate. Concomitantly, we only looked at maternal distress and general parenting practices. Future research should consider the beliefs of other family members as well as specific parenting practices in certain areas (e.g., digital parenting). For example, Cao et al. [111] found that Chinese parents should need more guidance and support for digital parenting. Fourth, the study only explored parent-driven effects, namely the influence of mothers on children’s media use. In the future, child-driven effects should be considered and the impact of children’s media use on parent-child interaction should be explored. Due to the young age of preschool children, it is difficult for them to actively access electronic media at home. Children’s ability to use electronic devices may be related to their parents’ beliefs about the effects of media/screen time [112]. Future research should focus on parents’ positive and negative beliefs about media use, as well as the relationship between belief and children’s media use. Finally, not considering interactions limits our ability to fully understand the complex relationships between different variables, which may affect the interpretation and inference of the phenomena studied. Future research should examine the interactions between these related variables to gain a more comprehensive understanding.

## Conclusion

The current study looks into the role of parenting stress and general parenting practices in mediating the relationship between maternal COVID-19 distress and children’s problematic media use. During lockdowns, parents may struggle to balance their personal lives, careers, and parenting responsibilities, making it difficult to maintain good mental health and limit their children’s media use. When facing stressful events, parents should address their emotions and stress as soon as possible and improve their mindfulness through learning and training. Moreover, parents should establish screen-time rules and not compromise on these rules. It is best for parents to support their children by using adaptive parenting techniques such as effective communication and interaction and diverting their attention away from unrestricted and excessive media use.

## Data Availability

All data generated or analyzed during this study are included in this published article.

## Abbreviations

PMU: Problematic Media Use
IT-CPU: Interactional Theory of Childhood Problematic Media Use
CEFIS: COVID-19 Exposure and Family Impact Survey
PSI-SF: Parenting Stress Index-Short Form
PSDQ: Parenting Styles and Dimensions Questionnaire
PMUM: Problematic Media Use Measure scale

## References

[1] Gueron-Sela N, Shalev I, Gordon-Hacker A (2023). Screen media exposure and behavioral adjustment in early childhood during and after COVID-19 home lockdown periods [J]. Comput Hum Behav.

[2] Ozturk Eyimaya A, Yalcin Irmak A (2021). Relationship between parenting practices and children’s screen time during the COVID-19 pandemic in Turkey [J]. J Pediatr Nurs.

[3] Rideout VJ, Foehr UG, Roberts DF, Generation. M 2: media in the lives of 8-to 18-Year-Olds [J]. Henry J Kaiser Family Foundation; 2010.

[4] Sigman A (2012). Time for a view on screen time [J]. Arch Dis Child.

[5] Li M-Y, Wang S-Q, Zhang Y (2022). Enemy or Friend—the Moderation Efect of Educational Scren activities on the relationship betwen Scren Time and Emergent literacy Skils of Preschool children [J]. J Educational Stud.

[6] Dai Y-Y, Ye H-S, Bai Q. Study on the factors influencing the screen time of children aged 3–6 in Anhui Province during the COVID-19 [J]. Volume 43. JournalofAnhuiNormalUniversity(NaturalScience); 2020. pp. 607–12. 06.

[7] Domoff SE, Borgen AL, Radesky JS (2020). Interactional theory of childhood problematic media use [J]. Hum Behav Emerg Technol.

[8] Ophir Y, Rosenberg H, Tikochinski R (2021). What are the psychological impacts of children’s screen use? A critical review and meta-analysis of the literature underlying the World Health Organization guidelines [J]. Comput Hum Behav.

[9] Slater MD (2004). Operationalizing and analyzing exposure: the Foundation of Media effects Research [J]. Journalism Mass Communication Q.

[10] Langer SL, Crain AL, Senso MM (2014). Predicting child physical activity and screen time: parental support for physical activity and general parenting styles [J]. J Pediatr Psychol.

[11] Zhu M, Zhang A-H, Cao Z (2017). Investigation on the exposure status of electronic screen in preschool children and the influencing factors [J]. Maternal Child Health Care China.

[12] Council O (2016). Media and young minds [J]. Pediatrics.

[13] Cnnic. The 51st Statistical Report on China’s Internet Development [Z]. 2023.

[14] Kabali HK, Irigoyen MM, Nunez-Davis R (2015). Exposure and use of Mobile Media devices by Young children [J]. Pediatrics.

[15] Radesky JS, Weeks HM, Ball R et al. Young Children’s Use of smartphones and tablets [J]. Pediatrics, 2020, 146(1). 10.1542/peds.2019-3518 PMC7329252 32482771

[16] Kraut R, Patterson M, Lundmark V (1998). Internet paradox: a social technology that reduces social involvement and psychological well-being? [J]. Am Psychol.

[17] Zimmerman FJ, Christakis DA (2007). Associations between content types of early media exposure and subsequent attentional problems [J]. Pediatrics.

[18] Domoff SE, Harrison K, Gearhardt AN (2019). Development and validation of the problematic media use measure: a parent report measure of screen media addiction in children [J]. Psychol Pop Media Cult.

[19] Bronfenbrenner U. The ecology of human development: experiments by nature and design [M]. Harvard University Press; 1979.

[20] Emond JA, Tantum LK, Gilbert-Diamond D (2018). Household chaos and screen media use among preschool-aged children: a cross-sectional study [J]. BMC Public Health.

[21] Radesky JS, Peacock-Chambers E, Zuckerman B (2016). Use of Mobile Technology to calm upset children: associations with Social-Emotional Development [J]. JAMA Pediatr.

[22] Zhu H, Wei L, Niu P. The novel coronavirus outbreak in Wuhan, China [J]. Global health research and policy, 2020, 5: 1–3. 10.1186/s41256-020-00135-6 PMC7050114 32226823

[23] Yeung J. 180 million people impacted by China’s Covid lockdowns. Here’s what you need to know [Z]. 2022.

[24] He L. 3 reasons Shanghai’s lockdown matters to the global economy [Z]. Cable News Network Business; 2022.

[25] Counted V, Pargament KI, Bechara AO (2022). Hope and well-being in vulnerable contexts during the COVID-19 pandemic: does religious coping matter? [J]. J Posit Psychol.

[26] Qiu J, Shen B, Zhao M (2020). A nationwide survey of psychological distress among Chinese people in the COVID-19 epidemic: implications and policy recommendations [J]. Gen Psychiatr.

[27] Kazak AE, Alderfer M, Enlow PT (2021). COVID-19 exposure and family impact scales: factor structure and initial psychometrics [J]. J Pediatr Psychol.

[28] Li J, Zhai Y, Xiao B (2023). Maternal COVID-19 distress and Chinese Preschool Children’s problematic media use: a Moderated serial mediation model [J]. Psychol Res Behav Manag.

[29] Babore A, Trumello C, Lombardi L, et al. Mothers’ and children’s Mental Health during the COVID-19 pandemic lockdown: the mediating role of parenting stress [J]. Volume 54. Child Psychiatry & Human Development; 2023. pp. 134–46. 1. 10.1007/s10578-021-01230-6 PMC8379586 34417927

[30] Cronin S, Becher E, Christians KS et al. Parents and stress: Understanding experiences, context, and responses [J]. 2015.

[31] Brooks SK, Webster RK, Smith LE (2020). The psychological impact of quarantine and how to reduce it: rapid review of the evidence [J]. Lancet.

[32] Prime H, Wade M, Browne DT (2020). Risk and resilience in family well-being during the COVID-19 pandemic [J]. Am Psychol.

[33] Forner D, Leslie P, Aldaihani A (2022). Psychosocial distress in parents with children awaiting surgery during the COVID-19 pandemic [J]. Children.

[34] Meraya A, Syed M, Yasmeen A (2021). COVID-19 related psychological distress and fears among mothers and pregnant women in Saudi Arabia [J]. PLoS ONE.

[35] Huang Y, Zhao N (2020). Generalized anxiety disorder, depressive symptoms and sleep quality during COVID-19 outbreak in China: a web-based cross-sectional survey [J]. Psychiatry Res.

[36] Burdette H, Whitaker R, Kahn R, et al. Association of Maternal Obesity and depressive symptoms with television-viewing time in low-income Preschool children [J]. Volume 157. Archives of pediatrics & adolescent medicine; 2003. pp. 894–9. 10.1001/archpedi.157.9.894 12963595

[37] Bui NH, Cruickshank M, Mcaloon J (2022). Handheld devices: the barrier for parents with Mental Health Difficulties in Child outcomes [J]. J Child Fam stud.

[38] Duch H, Fisher EM, Ensari I (2013). Screen time use in children under 3 years old: a systematic review of correlates [J]. Int J Behav Nutr Phys Activity.

[39] Conners NA, Tripathi SP, Clubb R (2007). Maternal characteristics associated with television viewing habits of low-income preschool children [J]. J Child Fam stud.

[40] Zhang J, Madigan S, Browne D. Caregivers’ psychological distress, technology use, and parenting: the importance of a multidimensional perspective [J]. Comput Hum Behav, 2022, 134.

[41] Vernon-Feagans L, Willoughby M, Garrett-Peters P (2016). Predictors of behavioral regulation in kindergarten: Household chaos, parenting, and early executive functions [J]. Dev Psychol.

[42] Beyens I, Eggermont S (2014). Putting Young Children in Front of the television: antecedents and outcomes of parents’ Use of Television as a babysitter [J]. Communication Q.

[43] Ribner AD, Coulanges L, Friedman S (2021). Screen time in the Coronavirus 2019 era: International trends of increasing Use among 3-to 7-Year-old children [J]. J Pediatr.

[44] Browne NT, Snethen JA, Greenberg CS (2021). When pandemics collide: the impact of COVID-19 on childhood obesity [J]. J Pediatr Nurs.

[45] Abidin RR (1990). Introduction to the special issue: the stresses of parenting [J]. J Clin Child Psychol.

[46] Deater-Deckard K, Scarr S (1996). Parenting stress among dual-earner mothers and fathers: are there gender differences? [J]. J Fam Psychol.

[47] Liu D, Ren Y, Yan F et al. Psychological impact and predisposing factors of the coronavirus disease 2019 (COVID-19) pandemic on general public in China [J]. 2020.

[48] Wang Y, Di Y, Ye J (2021). Study on the public psychological states and its related factors during the outbreak of coronavirus disease 2019 (COVID-19) in some regions of China [J]. Psychol Health Med.

[49] Del Boca D, Oggero N, Profeta P (2020). Women’s and men’s work, housework and childcare, before and during COVID-19 [J]. Rev Econ Househ.

[50] Pearlin LI. The sociological study of stress [J]. J Health Soc Behav, 1989: 241. 2674272

[51] Spinelli M, Lionetti F, Pastore M (2020). Parents’ stress and children’s psychological problems in families facing the COVID-19 outbreak in Italy [J]. Front Psychol.

[52] Marchetti D, Fontanesi L, Di Giandomenico S (2020). The effect of parent psychological distress on child hyperactivity/inattention during the COVID-19 lockdown: testing the mediation of parent verbal hostility and child emotional symptoms [J]. Front Psychol.

[53] Bolger N, Delongis A, Kessler RC et al. The contagion of stress across multiple roles [J]. J Marriage Fam, 1989: 175–83.

[54] Mcdaniel BT, Radesky JS, Technoference (2018). Longitudinal associations between parent technology use, parenting stress, and child behavior problems [J]. Pediatr Res.

[55] Radesky JS, Kistin C, Eisenberg S (2016). Parent perspectives on their Mobile Technology Use: the excitement and exhaustion of parenting while connected [J]. J Dev Behav Pediatr.

[56] Bandura A, Walters RH. Social learning theory [M]. Englewood cliffs Prentice Hall; 1977.

[57] Da Rosa LC, Pedrotti BG, Mallmann MY (2020). O papel da coparentalidade e da rede de apoio materna no uso de mídias digitais por bebês [J]. Contextos Clínicos.

[58] Carroll N, Sadowski A, Laila A (2020). The impact of COVID-19 on health behavior, stress, financial and food security among middle to high income Canadian families with young children [J]. Nutrients.

[59] Warren R, Aloia L, Parenting, Style (2019). Parental stress, and mediation of children’s media use [J]. Western J Communication.

[60] Baydar N, Akçınar B, İme N, Çevre. sosyoekonomik bağlam ve ana babalık [Environment, socioeconomic dependence and parenthood] [J]. Ana babalık: Kuram ve araştırma, 2012: 15 – 8.

[61] Kahraman H, Irmak TY, Basokcu TO. Parenting practices Scale: its validity and reliability for parents of school-aged children [J]. Volume 17. Educational Sciences-Theory & Practice; 2017. pp. 745–69. 3.

[62] Agnew R (1992). Foundation for a general strain theory of crime and delinquency [J]. Criminology.

[63] Belsky J (1993). Etiology of child maltreatment: a developmental-ecological analysis [J]. Psychol Bull.

[64] Pereda N, Díaz-Faes DA (2020). Family violence against children in the wake of COVID–19 pandemic: a review of current perspectives and risk factors [J]. Child Adolesc Psychiatry Mental Health.

[65] Le YY, Fredman SJ, Feinberg ME (2017). Parenting Stress Mediates the Association between Negative Affectivity and harsh parenting: a longitudinal dyadic analysis [J]. J Fam Psychol.

[66] Oliveira TDO, Costa DS, Alvim-Soares A (2022). Children’s behavioral problems, screen time, and sleep problems’ association with negative and positive parenting strategies during the COVID-19 outbreak in Brazil [J]. Child Abuse Negl.

[67] Li X, Hao C (2019). The relationship between parental attachment and mobile phone dependence among Chinese rural adolescents: the role of alexithymia and mindfulness [J]. Front Psychol.

[68] Lee EJ, Kim H (2018). Gender Differences in Smartphone Addiction Behaviors Associated with parent–child bonding, parent–child communication, and parental mediation among Korean Elementary School students [J]. J Addictions Nurs.

[69] Yue XD, Cheung C-K, Wong D. Addictive internet use and Parenting Patterns among Secondary School Students in Guangzhou and Hong Kong [J]. J Child Fam stud, 2014, 24.

[70] Abidin R. The determinants of parenting behavior [J]. Journal of clinical child and adolescent psychology -. J CLIN CHILD ADOLESC PSYCHOL. 1992;21:407–12.

[71] Loades ME, Chatburn E, Higson-Sweeney N (2020). Rapid systematic review: the impact of social isolation and loneliness on the Mental Health of Children and adolescents in the Context of COVID-19 [J]. J Am Acad Child Adolesc Psychiatry.

[72] Cuartas J (2020). Heightened risk of child maltreatment amid the COVID-19 pandemic can exacerbate mental health problems for the next generation [J]. Psychol Trauma.

[73] Griffith AK (2022). Parental burnout and child maltreatment during the COVID-19 pandemic [J]. J Fam Violence.

[74] Chung G, Lanier P, Wong PYJ (2022). Mediating effects of parental stress on harsh parenting and parent-child relationship during coronavirus (COVID-19) pandemic in Singapore [J]. J Fam Violence.

[75] Williamson JA, Mccabe JE, O’hara M W et al. Parenting stress in early motherhood: stress spillover and social support1 [J]. Compr Psychol, 2013, 2(1): 10.21. CP. 2.11.

[76] Neppl TK, Senia JM, Donnellan MB (2016). Effects of economic hardship: testing the family stress model over time [J]. J Fam Psychol.

[77] White RM, Liu Y, Nair RL (2015). Longitudinal and integrative tests of family stress model effects on Mexican origin adolescents [J]. Dev Psychol.

[78] Wozniak-Prus M, Gambin M, Sekowski M et al. Positive experiences in the parent-child relationship during the COVID-19 lockdown in Poland: the role of emotion regulation, empathy, parenting self-efficacy, and social support [J]. Fam Process, 2023: e12856. 10.1111/famp.12856 36724769

[79] Brazendale K, Beets MW, Weaver RG (2017). Understanding differences between summer vs. school obesogenic behaviors of children: the structured days hypothesis [J]. Int J Behav Nutr Phys Activity.

[80] Bate J, Pham PT, Borelli JL (2021). Be my safe haven: parent-child relationships and Emotional Health during COVID-19 [J]. J Pediatr Psychol.

[81] Enlow PT, Phan T-LT, Lewis AM (2022). Validation of the COVID-19 exposure and family impact scales [J]. J Pediatr Psychol.

[82] Eisinga R, Grotenhuis M, Pelzer B (2013). The reliability of a two-item scale: Pearson, Cronbach, or Spearman-Brown? [J]. Int J Public Health.

[83] Abidin R, Flens JR, Austin WG. The parenting stress index [M]. Lawrence Erlbaum Associates; 2006.

[84] Wang Y-J, Zhang M-X, Zhu J-J (2020). Maternal parenting stress and Preschooler’s Social competence: Mediating effects of parenting style [J]. Chin J Clin Psychol.

[85] Robinson CC, Mandleco B, Olsen SF et al. The parenting styles and dimensions questionnaire (PSDQ) [J]. Handbook of family measurement techniques, 2001, 3: 319 – 21.

[86] Wu P, Robinson C, Yang C (2010). Similarities and differences in mothers’ parenting of preschoolers in China and the United States [J]. Int J Behav Dev.

[87] Xiao B, Zhu L, Kong X, et al. Shyness and Socio-Emotional Adjustment in Early Childhood in Mainland China: exploring the roles of maternal parenting practices and beliefs about shyness [J]. J Child Fam Stud; 2022.

[88] Li J, Wang J, Xiao B et al. Translation and validation of the Chinese Version of the problematic media use measure [J]. Early Educ Dev, 2023: 1–16.

[89] Halpin S, Mitchell AE, Baker S et al. Parenting and child behaviour barriers to managing screen Time with Young children [J]. J Child Fam stud, 2021: 824–38.

[90] Park S, Chang HY, Park EJ (2018). Maternal depression and children’s screen overuse [J]. J Korean Med Sci.

[91] Giannotti M, Mazzoni N, Bentenuto A (2022). Family adjustment to COVID-19 lockdown in Italy: parental stress, coparenting, and child externalizing behavior [J]. Fam Process.

[92] Di Giorgio E, Di Riso D, Mioni G, et al. The interplay between mothers’ and children behavioral and psychological factors during COVID-19: an Italian study [J]. Volume 30. European child & adolescent psychiatry; 2021. pp. 1401–12. 9. 10.1007/s00787-020-01631-3 PMC7456665 32865654

[93] Tang L, Hruska V, Ma DWL et al. Parenting under pressure: stress is associated with mothers’ and fathers’ media parenting practices in Canada [J]. J Child Media, 2021: 233–48.

[94] Jiao WY, Wang LN, Liu J (2020). Behavioral and emotional disorders in children during the COVID-19 epidemic [J]. J Pediatr.

[95] Ma C-H, Jiang L, Chu L-T et al. Mental health problems of preschool children during the COVID-19 home quarantine: a cross-sectional study in Shanghai, China [J]. Front Psychol, 2022, 13. 10.3389/fpsyg.2022.1032244 PMC9665113 36389448

[96] Parks EP, Kazak A, Kumanyika S, et al. Perspectives on stress, parenting, and children’s obesity-related behaviors in black families [J]. Volume 43. Health Education & Behavior; 2016. pp. 632–40. 6. 10.1177/1090198115620418 PMC4935644 26733488

[97] Nabi RL, Krcmar M (2016). It takes two: the effect of child characteristics on U.S. parents’ motivations for allowing electronic media use [J]. J Child Media.

[98] Radesky JS, Silverstein M, Zuckerman B et al. Infant self-regulation and early Childhood Media exposure [J]. Pediatrics, 2014: e1172–8. 10.1542/peds.2013-2367 PMC4006432 24733868

[99] Padilla-Walker LM, Coyne SM, Collier KM (2016). Longitudinal relations between parental media monitoring and adolescent aggression, prosocial behavior, and externalizing problems [J]. J Adolesc.

[100] Romero E, Lopez-Romero L, Dominguez-Alvarez B (2020). Testing the effects of COVID-19 confinement in Spanish Children: the role of parents’ distress, emotional problems and specific parenting [J]. Int J Environ Res Public Health.

[101] Cimino S, Marzilli E, Tambelli R (2021). Psychological distress due to COVID-19 in parents and children’s emotional and conduct problems: the mediation role of couple adjustment and parenting stress [J]. Psychol Hub.

[102] Shin E, Choi K, Resor J (2021). Why do parents use screen media with toddlers? The role of child temperament and parenting stress in early screen use [J]. Infant Behav Dev.

[103] Wu Q, Xu Y (2020). Parenting stress and risk of child maltreatment during the COVID-19 pandemic: a family stress theory-informed perspective [J]. Dev Child Welf.

[104] Hester M, He JP, Tian L (2009). Girls’ and boys’ experiences and perceptions of parental Discipline and punishment while growing up in China and England [J]. Child Abuse Rev.

[105] Anthony LG, Anthony BJ, Glanville DN (2005). The relationships between parenting stress, parenting behaviour and preschoolers’ social competence and behaviour problems in the classroom [J]. Infant Child Development: Int J Res Pract.

[106] Crnic K, Low C. Everyday stresses and parenting [J]. 2002.

[107] Arim RG, Shapka JD (2008). The impact of pubertal timing and parental control on adolescent problem behaviors [J]. J Youth Adolesc.

[108] Mistry RS, Lowe ED, Benner A (2008). Expanding the family economic stress model: insights from a mixed-methods approach [J]. J Marriage Fam.

[109] Ponnet K, Financial, Stress (2014). Parent functioning and adolescent Problem Behavior: an actor–Partner Interdependence Approach to Family stress processes in Low-, Middle-, and high-income families [J]. J Youth Adolesc.

[110] Scrimin S, Mastromatteo LY, Hovnanyan A (2022). Effects of Socioeconomic Status, parental stress, and Family Support on children’s Physical and Emotional Health during the COVID-19 pandemic [J]. J Child Fam stud.

[111] Cao SM, Dong C a, Li M. H. Digital parenting during the COVID-19 lockdowns: how Chinese parents viewed and mediated young children’s digital use [J]. Early Child Dev Care, 2021: 1–16.

[112] Hefner D, Knop K, Schmitt S et al. Rules? Role model? Relationship? The impact of parents on their children’s problematic Mobile phone involvement [J]. Media Psychol, 2019: 82–108.

